# Nurse graduates’ experiences and support needs: A qualitative systematic review of South Africa’s community service programme

**DOI:** 10.4102/curationis.v42i1.1906

**Published:** 2019-08-21

**Authors:** Rita O. Abiodun, Felicity Daniels, Christoph Pimmer, Jennifer Chipps

**Affiliations:** 1 School of Nursing, University of the Western Cape, Cape Town, South Africa; 2 Department of Business Administration, University of Applied Sciences and Arts Northwestern Switzerland, Basel, Switzerland

**Keywords:** transition, nurse graduates, qualitative systematic review, community nursing service, South Africa

## Abstract

**Background:**

The student-to-nurse graduate transition is a pivotal phase in the professional development of nurses. In South Africa, this transition is part of a compulsory community service programme, which requires newly graduated nurses to work in rural and/or underserved areas for a period of 1 year.

**Objectives:**

The aim of this study was to review nurse graduates’ experiences and support needs during their transition in the compulsory community service programme.

**Methods:**

A qualitative systematic review of experiences was conducted. Qualitative research studies that addressed nurses’ experience in South Africa (2008–2017) were identified in Cochrane, Joanna Briggs Institute (JBI), Academic Search Complete, CINAHL, PubMed, SABINET, Science Direct, SCOPUS and Google Scholar databases. The systematic review methods included searching, sifting, abstracting and quality assessment of relevant qualitative studies by two reviewers and cross-checking by a third reviewer. Two reviewers independently performed blinded data extraction and quality assessment using the confidence in qualitative synthesis findings (ConQual) approach.

**Results:**

A total of 1257 studies were identified of which 12 met the inclusion criteria. Seven of the 12 studies were published articles and six were theses. The quality of the studies was found to be of high standard based on the ConQual rating. Four main themes emerged from the analysis: (1) rich developmental experiences through practice exposure, (2) difficulties in reconciling theory and practice, (3) contextual challenges in the workplace and (4) need for professional support structures, educational measures and public guidelines.

**Conclusion:**

Although positive experiences were reported, various challenges emerged, indicating the need for more systematic support mechanisms during transition.

## Introduction and background

In 1998, South Africa introduced a community service programme (CSP) for selected groups of health professionals after the completion of their training. This initiative aimed at promoting equity in the delivery of health services through the provision of human resources in rural and/or underserved areas with the long-term goal of retaining these health professionals to work in rural areas (SANC 2010). In 2008, community service was extended to include newly qualified nurses following the proclamation of the *Nursing Act* No. 33 of 2005 (SANC 2010). This meant that newly qualified professional nurses were required to serve in rural and/or underserved areas for a period of 12 months after completing their undergraduate degree or diploma in nursing. During the 12 months of community service, newly qualified nurses are registered as Community Service Nurse Practitioner (CSNP), after which they are registered as professional nurses with the South African Nursing Council (SANC 2010).

From a professional development perspective, this transition is central because it strongly impacts the development of skills and even future career success of nurses (Koen, Klehe & Van Vianen 2012). It can offer numerous opportunities for nurses to further develop their clinical skills, ethical and professional behaviour and their critical thinking abilities upon graduation (Hatcher et al. 2014). Community service provides a bridge to help students to make the transition from a well-structured education environment to more flexible self-guided workplace learning, where they assume greater responsibilities for their own learning and decision-making.

However, studies that explore this programme indicate that working in rural and marginalised settings is challenging. Having limited practice experience, newly qualified nurses are often placed in clinics in remote areas in which they are sometimes the most senior nurses (Thopola, Kgole & Mamogobo 2013). They experience high levels of stress, anxiety and feelings of isolation (D’ambra & Andrews 2014; Hlosana-Lunyawo & Yako 2013). According to Meleis (2015) and Zaayman (2016), the transition from students to nurse graduate and the responsibilities attached to it may lead to a state of disequilibrium.

Although a number of research studies have been conducted in South Africa on the transition experiences of student nurses into new graduates through the CSP, a systematic analysis of their experiences and support needs is still lacking. From a practical standpoint, the CSP does not yet have harmonised support mechanisms in place. This means that structures and practices vary considerably, depending on the local and institutional settings in which the CSNPs are placed. Accordingly, the experiences of CSNPs can manifest differently across different settings. This review seeks to address this gap by providing a synthesis of experiences to understand the needs of CSNPs and inform policies and strategies for support mechanisms during the transition period. This will be achieved by contrasting and synthesising the different experiences and discussing them in light of international literature.

## The review methods

### Aim

The aim of the study was to review nurse graduates’ experiences and support needs during transition in the compulsory CSP in South Africa.

### Research question

What are the experiences and support needs of graduate nurses in their transition to CSNPs in South Africa’s CSP?

### Design

A systematic review was conducted using the Joanna Briggs Institute (JBI) standard search procedures (Joanna Briggs Institute 2011). The approach summarises individual studies, critiques their design and makes recommendations for future research and practice.

### Search strategy

The search included published articles, following the guidelines of the JBI (2011), and also grey or unpublished literature on the compulsory community service in South Africa (2008–2017). A three-step search strategy was employed in the study. The search terms included ‘newly graduated nurse’, ‘community service nurse’, ‘South Africa’, ‘experience’, ‘needs’ and ‘perception’. The search was restricted to English language studies and was completed in December 2017. An initial search of Cochrane and JBI databases for published systematic reviews in this context was conducted by reviewing titles and abstracts, index and MeSH terms to ensure that all relevant materials were captured. A second search with the identified keywords and index terms was undertaken using the following electronic databases: Cochrane, JBI, Academic Search Complete, CINAHL, PubMed, SABINET, Science Direct, SCOPUS and Google Scholar. Thirdly, the reference lists of identified articles were back-searched. The identified studies were assessed for relevance based on the title and, where available, the abstract. The full article was retrieved and further assessed by two reviewers based on the inclusion criteria. All full texts were analysed against three inclusion criteria (see Table 1). The search strategy was summarised using the PRISMA statement flow chart (Moher, Liberati, Tetzlaff & Altman 2009).

**TABLE 1:** Inclusion and exclusion criteria.

Variable	Description
Population	Inclusion: Newly qualified nurses (community service nurses) during their year of compulsory community service in South Africa.
	Exclusion: Registered nurses in their first year of practice after the compulsory community service.
Phenomenon of interest or outcome	Experiences (positive and negative), support needs and attendant strategies during their year of compulsory community service in South Africa were the phenomena of interest.
Type of study	Qualitative studies, not limited to design or publication type.

### Critical appraisal (assessment of methodological quality)

The confidence in qualitative (ConQual) approach was used to assess the methodological quality of the study. This approach includes two major elements that influence the confidence of qualitative synthesised findings: (1) dependability (a measure of trustworthiness that helps to establish whether a study’s findings are consistent and repeatable) and (2) credibility (a measure of trustworthiness that helps researchers to link a study’s findings to reality) (Munn et al. 2014).

All qualitative studies selected for retrieval were assessed independently by two reviewers for methodological quality and cross-checked by a third reviewer before inclusion in the review. Any disagreements between the reviewers were resolved through discussion (Munn et al. 2014). Dependability was determined to be high, moderate, low or very low based on the following criteria: (1) congruence between the research methodology and the research question or objectives, (2) congruence between the research methodology and the methods used to collect data, (3) congruence between the research methodology and the representation and analysis of data, (4) statements that located the researcher culturally or theoretically and (5) description of the influence of the researcher on the research (and vice versa). Credibility was appraised as either unequivocal (findings accompanied by an illustration that is beyond reasonable doubt and therefore not open to challenge), equivocal (findings accompanied by an illustration lacking clear association with it and therefore open to challenge) or unsupported or mixed (findings are not supported by the data). In addition, the reviewers’ judgements on the relevance and appropriateness of each study and its methodology were considered.

### Data extraction

Qualitative data were extracted using a standardised data extraction tool: the JBI qualitative assessment and review instrument (JBI-QARI). Two reviewers independently extracted the data and any disagreements were resolved by discussion. The data extracted included the demographic information of the population, the phenomena of interest, study methods and findings that were relevant to the research question.

### Data analysis

Thematic analysis was employed in this study because it offers a systematic approach to organise, analyse and present qualitative data collected from different sources of literature (Thomas & Harden 2008).

Thematic analysis was used to categorise the experiences and support needs of newly qualified nurses, which were grouped together into meaningful themes.

The analytical process involved the reading and re-reading of the studies before extracting relevant statements (Thomas & Harden 2008). The extracted content was reviewed iteratively against the emerging themes, which were inferred with constant recourse to the research question on experiences and support needs of newly graduated nurses. The analysis yielded 35 categories which were then grouped into 4 main themes (see Table 2).

**TABLE 2:** Themes, sub-themes and categories of experiences of new community service nurses.

Variable	Descriptions
Theme 1	Rich developmental experiences and confidence development (through practice exposure)
Sub-theme 1.1	Developmental experience
Categories	Time to grow and develop professionally and role development (Andrén & Hammami 2011; Plessis & Seekoe 2013; Thopola et al. 2013). Gained from being exposed to different institutional settings (Govender et al. 2017). Working with patient and family as a satisfying experience (Govender et al. 2017).
Sub-theme 1.2	Development of confidence through practice exposure
Categories	Sense of achievement and confidence (Roziers et al. 2014). Development of communication skills and relationships (Ndaba & Nkosi 2015; Zaayman 2016).
Theme 2	Difficulties in theory and practice
Sub-theme 2.1	Theory to practice praxis
Categories	Challenges in the integration of theory and practice (Tsotetsi 2013; Zaayman 2016). Realities of practice: reality shock (Ndaba & Nkosi 2015; Roziers et al. 2014). Ambiguous situations challenging (Govender et al. 2017; Plessis & Seekoe 2013).
Sub-theme 2.2	Poor practice readiness, anxiety and stress
Categories	Feeling unprepared from school (Beyers 2013; Nkoane 2015). Poor practice readiness (Nkoane 2015). Anxiety and stress (Ndaba & Nkosi 2015; Plessis & Seekoe 2013; Roziers et al. 2014; Shezi 2014).
Theme 3	Contextual challenges in the work place
Sub-theme 3.1	Lack of organisational or supervision support
Categories	Lack of orientation (Govender et al. 2017; Ndaba & Nkosi 2015; Thopola et al. 2013). Lack of team work (Andrén & Hammami 2011; Thopola et al. 2013). Limited support (Beyers 2013; Ndaba & Nkosi 2015; Tsotetsi 2013). Lack of supervision and mentorship (Nkoane 2015; Thopola et al. 2013). Uncertainty with unclear objectives of the community service policy (Govender et al. 2017; Thopola et al. 2013).
Sub-theme 3.2	Restricted human and material resources and high workload
Categories	Lack of human and material resources in practice settings (Ndaba & Nkosi 2015; Nkoane 2015; Thopola et al. 2013). High workloads (Andrén & Hammami 2011; Plessis & Seekoe 2013; Shezi 2014; Tsotetsi 2013). Low remuneration (Andrén & Hammami 2011; Govender et al. 2017; Thopola et al. 2013; Tsotetsi 2013; Nkoane 2015).
Sub-theme 3.3	Interpersonal challenges (difficult relationships)
Categories	Bullying and strife among staff (Beyers 2013; Ndaba & Nkosi 2015; Tsotetsi 2013). Poor communication between staff (Tsotetsi 2013). Poor acceptance of new nurses (Govender et al. 2017). Loneliness (Beyers 2013).
Theme 4	Need for professional support structures, measures and public guidelines
Sub-theme 4.1	Need for professional structures and measures
Categories	Well-structured orientation programme (Beyers 2013; Govender et al. 2017; Nkoane 2015; Roziers et al. 2014; Shezi 2014; Tsotetsi 2013; Zaayman 2016). Coaching and mentorship (Beyers 2013; Govender et al. 2017; Hlosana-Lunyawo & Yako 2013; Plessis & Seekoe 2013; Shezi 2014; Tsotetsi 2013; Zaayman 2016). Creating call centres for support (Nkoane 2015; Tsotetsi 2013). Communication of evidence-based information to inform practice (Roziers et al. 2014; Plessis & Seekoe 2013). Continuous professional development (CPD) (Nkoane 2015; Thopola et al. 2013; Tsotetsi 2013; Zaayman 2016).
Sub-theme 4.2	Educational or training issues
Categories	Well-structured curriculum for proper preparation of nurses (Andrén & Hammami 2011; Tsotetsi 2013; Zaayman 2016). Inappropriate induction programmes (Hlosana-Lunyawo & Yako 2013). Inadequate training programmes during preparation (Hlosana-Lunyawo & Yako 2013; Zaayman 2016).
Sub-theme 4.3	Need for guidelines for Community Service Practice
Categories	Guidelines for community service policy stakeholders (Nkoane 2015; Tsotetsi 2013; Zaayman 2016). Guidelines for supervision (Hlosana-Lunyawo & Yako 2013; Nkoane 2015; Tsotetsi 2013). Guidelines for support (Andrén & Hammami 2011; Hlosana-Lunyawo & Yako 2013; Nkoane 2015; Shezi 2014; Tsotetsi 2013). Guidelines for role clarification (Andrén & Hammami 2011; Shezi 2014; Tsotetsi 2013).

### Ethical considerations

Ethical approval was obtained from the University of the Western Cape Research Ethics Committee (HS16/6/41).

## Results and discussions

### Search results

**FIGURE 1:**
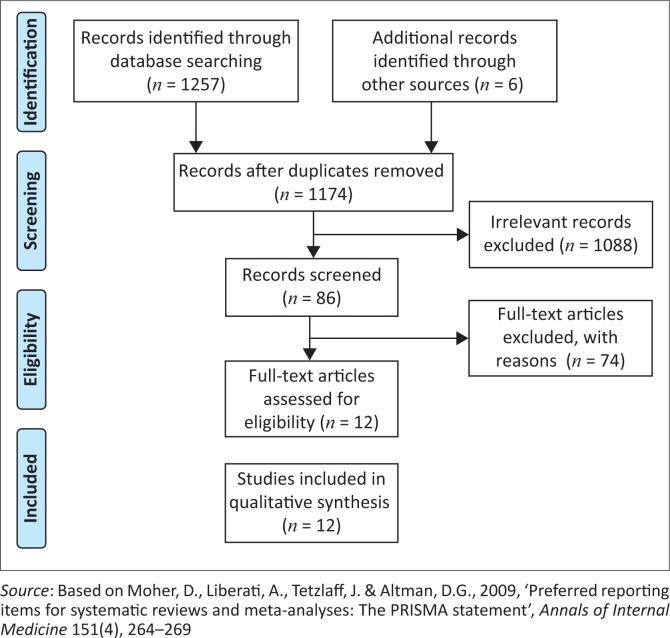
Prisma flow diagram.

The systematic search identified a total of 1263 studies of which 1257 were extracted from the initial search and 6 from grey literature (Figure 1). Duplicates and irrelevant studies were removed by the review of titles. As a result, 86 studies that potentially met the inclusion criteria remained. Thereafter, a detailed review of the title and abstract of each study was performed, resulting in the final number of 12 studies. The 12 studies included seven full text articles and six unpublished theses that met the inclusion criteria. No additional studies were identified from the reference lists of these articles.

### Quality of studies

The 12 studies that met the inclusion criteria (see Table 1) were reviewed and critically appraised. Seven studies were determined to have high quality (Beyers 2013; Govender, Brysiewicz & Bhengu 2017; Hlosana-Lunyawo & Yako 2013; Nkoane 2015; Plessis & Seekoe 2013; Roziers, Kyriacos & Ramugondo 2014; Zaayman 2016), five studies were of moderate quality (Andrén & Hammami 2011; Ndaba & Nkosi 2015; Shezi 2014; Thopola et al. 2013; Tsotetsi 2013) and one study was evaluated to be of low quality and was subsequently removed.

### Description of selected studies

Of the 12 qualitative research studies selected for inclusion, six were published in peer-reviewed journals (Govender et al. 2017; Hlosana-Lunyawo & Yako 2013; Ndaba and Nkosi 2015; Plessis and Seekoe 2013; Roziers et al. 2014; Thopola et al. 2013) and six were unpublished theses (Andrén & Hammami 2011; Beyers 2013; Nkoane 2015; Shezi 2014; Tsotetsi 2013; Zaayman 2016). Eleven of the studies focused on experiences and one study centred explicitly on the needs of community service nurses (Shezi 2014). The studies were conducted in five provinces of South Africa (Eastern Cape, Gauteng, KwaZulu-Natal, Limpopo and Western Cape). The 12 qualitative studies included various designs. Six studies used a phenomenological approach (Hlosana-Lunyawo & Yako 2013; Ndaba & Nkosi 2015; Roziers et al. 2014; Plessis & Seekoe 2013; Shezi 2014; Thopola et al. 2013), whereas the remaining studies used descriptive-exploratory designs (see Table 3) (Andrén & Hammami 2011; Beyers 2013; Govender et al. 2017; Shezi 2014; Tsotetsi 2013; Zaayman 2016). The participants in the studies were all CSNPs, with the total number of participants in each study varying between 7 and 27. The predominant data collection tool was the individual interview.

**TABLE 3:** Data extraction and quality assessment.

Author (year)	Population and number (*n*) of participants	Phenomenon of interest	Design, methods and analysis	Findings	Overall ConQual score
Andrén and Hammami 2011	Newly qualified nurses 7 participants	Experiences of newly qualified nurses of University of Limpopo, executing mandatory community service in Limpopo province, South Africa.	A qualitative, exploratory, descriptive and contextual design. Qualitative content analysis.	**Experiences** Low salary. Lack of material resources. Shortage of staff. Lack of organisation of management. Missing parts in the structure of curriculum. Opportunities to develop within the profession. **Needs or strategies** Guideline for support.	Moderate *Dependability* – Downgrade 1 level† *Credibility* – No downgrade
Tsotetsi 2013	Newly qualified 4-year trained professional nurses 6–10 participants each	Experiences and support of the newly qualified 4-year trained professional nurses placed for remunerated community service in Gauteng province.	A qualitative, exploratory, descriptive and contextual study design. Five focus groups. *Tesch’s method of data analysis*	**Experiences** Mixed experiences. Adequate, inadequate, incidental and lack of support. Difficulty in the integration of theory into practice. Bad staff attitudes. Severe staff shortage. Poor interpersonal relationship and low salary. Role conflicts. **Needs or strategies** Standardised orientation programme. Coaching and mentorship. Role clarification or structured programme or scope of practice for community service. Incentives and resources. Review of curriculum in line with policies developed by DoH. Call centre for CSNs. Availability of policy guidelines. Pocket procedure manual with simple policies and protocols.	Moderate *Dependability* – Downgrade 1 level† *Credibility* – No downgrade
Plessis and Seekoe 2013	Newly qualified midwives	Experiences and challenges of newly qualified midwives in community services.	A qualitative explorative phenomenological design. Thematic analysis using Tesch’s method.	**Experiences** Positive experiences-training added value to clinical and professional experience. Sense of disillusionment. Stress (interpersonal relationships) in the clinical field. **Needs or strategies** Guidelines and policies for orientation and mentorship. Conduct in-service training session and continued professional development. Strategies to improve effective communication among staffs should be employed. Communication of evidence-based information to inform practice.	High *Dependability* – No downgrade *Credibility* – No downgrade
Thopola et al. 2013	Newly qualified nurses 8 participants	Experiences of newly qualified nurses at University of Limpopo, Turfloop campus executing community services in Limpopo province, South Africa.	A qualitative, explorative, descriptive and contextual research design with a phenomenological approach was adopted. Tesch’s content analysis.	**Experiences** Poor orientation. Lack of supervision. Lack of team work. Low remuneration. Shortage of human resources. Training added value to their competence. **Needs or strategies** Orientation programme. In-service education. Remuneration policy should be revisited.	Moderate *Dependability* – Downgrade 1 level† *Credibility* – No downgrade
Hlosana-Lunyawo and Yako 2013	Newly qualified professional nurses 20 participants	Experiences of newly qualified professional nurses in primary health care facilities in the Amathole District, Eastern Cape Province, South Africa.	A qualitative and exploratory approach. Using phenomenological design. Coliazzi’s six steps of data analysis.	**Experiences** Inappropriate induction programmes. Lack of support and supervision. Inadequate training and development programmes. **Needs or strategies** Guidelines for induction programmes, with role clarification. Guidelines on supervision. Coaching and mentoring programme.	High *Dependability* – No downgrade *Credibility* – No downgrade
Beyers 2013	Community service practitioners 10 participants	Experiences of community service practitioners who are deployed at a rural health facility in the Western Cape.	A qualitative, exploratory and descriptive design. The process of inductive coding of Thomas (2003) was used to analyse the data.	**Experiences** Challenges related to adaptation to a new environment (under preparedness, interpersonal relationships, professional accountability, lack of support from management). Positive experiences (boosted confidence and creativity and interpersonal support). **Needs or strategies** Timely orientation. Structured policy for community service. Mentor and preceptorship workshop.	High *Dependability* – No downgrade *Credibility* – No downgrade
Shezi 2014	Community service nurses 12 participants	The needs of community service nurses with regard to supervision and clinical accompaniment.	A qualitative design using exploratory, descriptive and contextual strategies. Qualitative content analysis.	**Experiences** Negative emotional experiences such as stress, fear, frustration, grievances and disorientation. Shortage of staff. Constructive support from experienced professional nurses. **Needs or strategies** A strategic plan for community service nurses: Induction and orientation, policies and procedures to be in place. Clinical supervision should be provided. Constructive support and emotional support should be provided. Sensitive feedback should be provided. Experienced professional nurses with positive attitude within the units. Job description of the community service nurse.	Moderate *Dependability* – No downgrade *Credibility* – Downgrade 1 level†
Roziers et al. 2014	Newly qualified South African nurses 8 participants	Newly qualified South African nurses’ lived experience of transition from student-to-community Service nurse.	Descriptive phenomenology (Husserl’s): Two semi-structured individual interviews conducted 6 weeks apart, before and after placement. Thematic data analysis.	**Experiences** Sense of achievement. Uncertainty and fear in anticipation of reality. Reality shock. **Needs or strategies** Structured programme. Guided realistic clinical scenarios during training.	High *Dependability* – No downgrade *Credibility* – No downgrade
Nkoane 2015	Community service nurses 11 participants	Community service nurses’ experiences regarding health care services at Tshwane district public hospital.	A qualitative interpretative phenomenological analysis (IPA) approach. 11 semi-structured interviews. Interpretative Phenomenological analysis framework for data analysis.	**Experiences** Shortage of material resources and human resources. Non-payment of allowances. Poor attitude. Lack of professional support. Poor practice readiness. Lack of motivation and confidence. Frustration. **Needs or strategies** In-service training and workshops. Psychological support. Guidelines for supervision and support. Availability of community service policy to all stakeholders.	High *Dependability* – No downgrade *Credibility* – No downgrade
Ndaba and Nkosi 2015	Qualified professional nurses 10 participants	Lived experiences of newly qualified professional nurses on community service in midwifery.	A qualitative descriptive, interpretive phenomenological research with interviews. Thematic analysis (Husserl’s philosophy of descriptive phenomenology).	**Experiences** Lack of orientation. Transition created a period of stress, uncertainty and fear which create emotional reactions. Positive (good communication) and negative (bullying and conflict) attitude from colleagues. **Needs or strategies** Proper orientation.	Moderate *Dependability* – No downgrade *Credibility* – Downgrade 1 level†
Zaayman 2016	Professional nurses 10 participants	Professional nurses’ experiences of their community service placement year at a secondary academic hospital in the Western Cape.	A qualitative research approach and an exploratory and descriptive research design. The process of inductive coding of Thomas (2003) was used to analyse the data.	**Experiences** Difficulty in applying new knowledge. Positive relationships with staff and some negative attitudes from staff. Experienced professional development. Inadequate undergraduate programme preparation. Experienced support and challenges (working alone, feeling of inexperience). **Needs or strategies** Nursing programme curriculum should be revisited. Jointly prepared guidelines by stakeholders. Long- and short-term support (revised orientation programme, debriefing opportunities, workshop and in-service training). Mentorship programme.	High *Dependability* – No downgrade *Credibility* – No downgrade
Govender et al. 2017	Nurses performing compulsory community service 27 participants	Pre-licensure experiences of nurses performing compulsory community service in KwaZulu-Natal, South Africa: A qualitative study.	A descriptive qualitative approach. Tesch’s content analysis. 27 semi-structured interviews.	**Experiences** Satisfied with allocation of sites. Gained from exposures. Feeling overwhelmed. Role confusion. Experiencing some frustration. **Needs or strategies** Proper orientation to CS. Scope of practice for CNPs should be developed. Proper remuneration should be reconsidered.	High *Dependability* – No downgrade *Credibility* – No downgrade

DoH, Department of Health; CSN, Community Service Nurse; CS, Community Service; CNP, Community service Nurse Practitioner.

† , Downgrading for dependability may occur when the five criteria for dependability are not met across the included studies.

### Synthesis

Thirty-five categories emerged from the analysis, which were grouped into four main themes (see Table 2). The four main themes are (1) rich developmental experiences and confidence development through practice exposure; (2) difficulties in reconciling theory and practice; (3) contextual challenges in the workplace and (4) need for professional support structures, measures and public guidelines.

## Discussion

### Theme 1: Rich developmental experiences and confidence gains through practice exposure

#### Developmental experiences

Five categories describe the rich developmental experiences of CSNPs through practice exposure and the training experienced during community service (see Table 2). A number of studies indicate that CSNPs consider the community service as a time to grow and to develop significant professional skills (Andrén & Hammami 2011; Plessis & Seekoe 2013; Thopola et al. 2013). A qualitative study by Thopola et al. (2013) reported that CSNPs emphasised personal and professional growth and development during the transition period. Newly graduated nurses acknowledged developmental opportunities, such as working with patients and their families, and other staff members; and in doing so, they advanced their communication skills. Govender et al. (2017) reported that it was rotation patterns and schedules which triggered professional growth and provided CSNPs with the opportunity to extend their skill set.

Similar observations can be found in international studies, which affirm the developmental effects associated with the student-to-nurse transition period. Ivey (2012) reports that newly graduated nurses in the United States had positive learning experiences during their year of transitioning, especially in situations in which they were welcomed and explicitly supported in their learning. Similarly, Harding et al. (2013) found that newly graduated nurses in the United States had an increase in knowledge and skills and that they advanced their professional development. To promote and sustain these effects, it has been argued that clinical sites should more explicitly acknowledge the developmental value of transitions, for example by increasing resources provided to new nurses to join membership organisations and by supporting them in the pursuit of certifications and further and specialised training (Walsh 2018).

#### Development of confidence through practice exposure

In addition to skills development, some studies also highlight that the CSP facilitated the development of a sense of achievement and confidence (Roziers et al. 2014). For example, Roziers et al. (2014) report that newly graduated nurses from the Western Cape province increased the confidence in their abilities to organise and prioritise duties, communicate with staff members and patients, and to assume more responsibilities and leadership roles.

Kumaran and Carney (2014) found that interpersonal and communication skills gained during the transition period increased the confidence of Irish nurse graduates. The confidence was developed through practice exposure in the transition period and it contributed to an enhanced self-image and higher levels of assertiveness. These factors may facilitate a smoother transition into new roles and prepare nurses to assume more responsibilities (Kumaran & Carney 2014). While Lea and Cruickshank (2015) described that new graduates generally enjoyed their rotations, these can also cause feelings of not belonging and may thus negatively impact their confidence.

### Theme 2: Difficulties in reconciling theory and practice

Theme 2 comprises two sub-themes that describe the difficulties that CSNPs face in reconciling theoretical knowledge with clinical practice (see Table 2).

#### Theory to practice praxis

Studies show that CSNPs faced considerable challenges in incorporating theoretical knowledge gained from their nursing education institution with practice experiences in the CSP (Tsotetsi 2013; Zaayman 2016).

Beyers (2013) and Nkoane (2015) state that difficulties of the reconciliation of theoretical and more practical knowledge are linked to CSNPs’ poor levels of practice readiness. Similarly, a qualitative study conducted by Nkoane (2015) in public hospitals reports that theoretical knowledge provided at nursing education institutions differs from the realities of clinical practice. Furthermore, the study reported that senior professional nurses do not provide adequate opportunities for CSNPs to apply their theoretical knowledge in clinical practice (Nkoane 2015). Also, this theme is corroborated by studies from outside of South Africa’s CSNP, saying that the school-to-work trajectory is very challenging because nurses struggle with the integration of theoretical knowledge with work practices (Feng & Tsai 2012; Ndaba & Nkosi 2015; Roziers et al. 2014).

#### Poor practice readiness, anxiety and stress

Some studies indicate that CSNPs experience anxiety, confusion and stress because of their inability to align theoretical knowledge from textbooks with the lived realities of clinical practice (Ndaba & Nkosi 2015; Plessis & Seekoe 2013; Roziers et al. 2014; Shezi 2014).

In the international literature, negative experience and low levels of practice readiness in the transition phase are linked to diverging role conceptions: A study on newly graduated nurses in Taiwan reports nurses experience difficulties in the differentiation of ‘idealised role conceptions’ and ‘actualised role conceptions’, causing anxiety, stress and a ‘reality shock’ (Cheng et al. 2014). In addition, Odetola et al. (2018) reported that the ‘disconnect’ between the theory and practice is one of the main reasons for professional fear, anxiety and feelings of incompetence among freshly graduated nurses.

There are suggestions in the literature about how to reduce or close the theory–practice gap which put nurse educators and mentors in the forefront. They can help new graduates by encouraging reflection (on-action) and critical thinking. Another suggestion from Swedish and Nigerian settings is that education should better prepare nursing students for the working life by offering more practical opportunities during their studies (Pennbrant et al. 2013). In the workplace, the support of academic–clinician collaboration, the building of a research culture and the improvement of research access to encourage the use of evidence-based studies to inform practice was suggested (Leach & Tucker 2018). However, the prerequisites for these measures are the proper staffing and the provision of resources to hospitals in rural and remote areas, which are often inadequate today.

### Theme 3: Contextual challenges in the work place

In addition to the theory–practice gap, which refers to tensions and contradictions between education institutions’ practise and practices of nurses’ (subsequent) working life, CSNPs experience an array of challenges relating to specific conditions within their workplaces. These challenges are classified according to three interdependent dimensions, namely (1) a lack of organisational and supervision structures, (2) restricted human and material resources and a high workload and (3) interpersonal challenges.

#### Lack of organisational and supervision structures

Several studies outline the lack of orientation, support, supervision and mentorship made available to CSNPs (Nkoane 2015; Thopola et al. 2013). For example, Govender et al. (2017) reported that CSNPs experienced limited orientation, mentoring and support as a result of staff shortages, which made the immersion into the workplace a stressful experience.

The lack of organisational or managerial support structures in the health care system has also been acknowledged in the wider literature. Although newly qualified nurses may initially feel excited at the onset of the transition, the lack of support structures can make newly acquired professional responsibilities an overwhelming experience (Kumaran & Carney 2014). Regan et al. (2017) reported that newly graduated nurses in Canada faced significant challenges in the immersion into their respective teams because of a lack of supportive mentors. The literature suggests that supportive relationships between graduates and their institutions need to develop over time in that autonomous practice and critical thinking (Hofler & Thomas 2016) are accompanied by the provision of adequate orientation and the availability of mentors and preceptors, and they can be supported through the use of information and communication technologies particularly in rural areas (Kaihlanen et al. 2018; Mbemba et al. 2013).

#### Restricted human and material resources and high workload

Several studies point to a deficit of human and material resources in clinical sites which is viewed as a particular challenge by nurses in the transition phase (Ndaba & Nkosi 2015; Nkoane 2015; Thopola et al. 2013). The lack of human resources accumulates in high workloads for the local staff, and particularly for the CSNPs, resulting in a number of negative consequences (Andrén & Hammami 2011; Plessis & Seekoe 2013; Shezi 2014). For example, Nkoane (2015) reports that the shortage of both human and material resources increased the workload of CSNPs which, in turn, resulted in high levels of stress and frustration.

The problem of high workload has also been identified in the literature beyond South Africa’s CSNP.

Several studies argued that high workload resulted in the experience of stress and uncertainty among newly graduated nurses (Blomberg et al. 2016; Higgins, Spencer & Kane 2010; Horsburgh & Ross 2013). For example, Regan et al. (2017) describe that the shortage of human resources in Canadian health care settings leads to heavy workloads of new nurses. This phenomenon is compounded by the inability of newly graduated nurses to effectively manage their workloads and their reliance on ‘self-generated’ approaches to address these issues (Bisholt 2012).

#### Interpersonal challenges

Several studies have pointed to interpersonal issues that CSNPs face in their relationships with nursing and clinical staff including poor communication, interpersonal problems, strife and bullying (Beyers 2013; Ndaba & Nkosi 2015;). A study from hospitals in Gauteng CSNPs reported poor communication and strife among staff, which were linked to restricted learning opportunities (Tsotetsi 2013). In addition, newly qualified nurses reported a sense of loneliness and isolation as a result of not feeling accepted at the workplace (Govender et al. 2017). Tsotetsi (2013) argues that the ability to connect socially with the new environment depends on the individual and can vary considerably, but some studies clearly identify unacceptable attitudes and behaviours from senior colleagues (D’ambra & Andrews 2014; Rush et al. 2014). Lea and Cruickshank (2015) identify negative attitudes and unsupportive behaviours of experienced nursing staff towards newly qualified nurses as key factors that jeopardise the success of the transition period. In addition, power imbalances among nursing and health professional teams further compound the problem with CSNPs being seen at the bottom of the ‘pecking order’ (Halpin, Terry & Curzio 2017).

Some nurses are reported to find it difficult to challenge the authority of superiors in the workplace and instead direct negative attitudes and frustration towards younger nurses and CSNPs (D’ambra & Andrews 2014). Against this backdrop, literature recommends managerial interventions and particularly the adoption of a participatory and dialogic leadership style, which promotes healthy relationships through social activities both in and outside of the work environment (Amestoy et al. 2014; Obakpolo 2015; Shah 2017).

### Theme 4: Need for professional support structures, educational measures and public guidelines

Theme 4 consists of three sub-themes: (1) the need for professional structures, (2) education and training measures and (3) the need for guidelines for the Community Service Practice (see Table 2). The eight subcategories that CSN needs are well-structured orientation programmes, coaching and mentorship; creation of call centres; communication of evidenced-based information to inform practice; and continuous professional development (CPD), a well-structured curriculum for proper preparation of nurses (Hlosana-Lunyawo & Yako 2013; Zaayman 2016).

#### The need for professional structures

There is wide agreement that newly graduated nurses require a period of structured support following their graduation (Al Awaisi, Cooke & Pryjmachuk 2015; Blomberg et al. 2016; Ebrahimi et al. 2016; Halpin et al. 2017; Ketelaar et al. 2015; Pasila, Elo & Kääriäinen 2017; Rush et al. 2014; Zamanzadeh et al. 2014). A number of studies outside of South Africa support this claim, saying that support mechanisms such as structured transitioning programmes facilitate newly graduated nurses in their nursing education institution-to-work trajectory (Baxter 2010; Park & Jones 2010; Parker, Cleveland & Ah-Thion 2014a; Pasila et al. 2017). High-quality programmes are necessary to provide reliable support structures for newly graduated nurses in the first year of practice (Whitehead et al. 2013). For example, the Nursing and Midwifery Council of United Kingdom recommends the development of globally accepted programmes of transition for a minimum period of 4 months to support newly graduated nurses (McCarthy & Murphy 2010). These programmes include continued informal group or peer support and mentoring (McCarthy & Murphy 2010).

There are particular aspects of practising nurses’ roles and responsibilities which require specific support during their transition into a new environment (Lea & Cruickshank 2015). Supportive strategies for newly graduated nurses include timely induction, appropriate orientation of new nurses, the provision of a supportive environment and authentic mentorship which is offered by seasoned nurses (Pineau et al. 2015; Regan et al. 2017). In addition, nursing leadership is vital in creating an organisational and unit or team culture that supports new nurses as they move along the transition continuum (Regan et al. 2017). A review conducted in the United States recommends several support strategies, such as internship, orientation programmes, mentorship and simulation-based graduate programmes to improve support structures of newly graduated nurses, which were found to be effective (Edwards et al. 2015). The value of these measures was confirmed by another integrative review on best practices of newly graduated nurse transition programmes (Rush et al. 2013).

#### Educational and training measures

Education and training play a pivotal role in the transition period. Firstly, inadequate prior training and preparation highlights the demand for more practice-orientated degree programmes (Hlosana-Lunyawo & Yako 2013; Zaayman 2016). For example, a study conducted in a nursing education institution in the Western Cape confirms that CSNPs feel that they are not adequately prepared for work because of a lack of practice exposure. The demand for more adequate, practice-centred education programmes is not restricted to the geographical context under investigation. For example, Azimian, Negarandeh and Fakhr-Movahedi (2014) report on factors that impact on Iranian nurses’ abilities to cope with transition. The study identified inappropriate educational preparation for practice settings as one of the key challenges faced by newly graduated nurses (Azimian, Negarandeh & Fakhr-Movahedi 2014). It can be recommended that nursing education institutions should develop curricula which are more practice-based and which would thus help nursing students build sufficient skills and confidence prior to the commencement of the community service period (Meleis 2015).

Secondly, there is also a need for training at the beginning of the service itself, for example in the form of properly structured induction programmes, such as extensive workshops which help CSPNs adapt to their new work environments more easily (Hlosana-Lunyawo & Yako 2013). After the induction, new nurses could be further accompanied through the provision of a well-structured evidence-based, clinical and professional education support programme that draws on their specific needs. These education programmes should, for example, include opportunities for reflective practice (Bull et al. 2015; Bulman 2013). In this respect, mobile technology can also be used, particularly in support of geographically distributed graduates.

A study from Nigerian settings explored WhatsApp groups in which moderators shared relevant knowledge and stimulated professional discussions over a period of 6 months following a predefined, needs-orientated curriculum. The newly graduated nurses had significantly higher knowledge and exhibited fewer feelings of professional isolation compared with a control group (Pimmer et al. 2019).

#### Need for guidelines for Community Service Practice

To harmonise and anchor measures in existing programmes, Rush et al. (2014) suggest guidelines for support opportunities for new graduates which include resource person(s), specific transition programmes for a period of at least 9 months (including educational measures). This would provide newly graduated nurses with substantial support during their transition period. In addition to these guidelines, Rush et al. also recommend the development of public guidelines and clear policies that describe the expectations and responsibilities of newly graduated nurses in the transition programme (Rush et al. 2013). Examples of such guidelines include those directed at community service policy stakeholders, guidelines for supervision, guidelines for support and guidelines to clarify the role of CSNPs (Rush et al. 2013).

## Limitations

Although this systematic review identified high-quality studies with relevant themes that were further bolstered by findings from studies outside of the scope of this review, there are a number of limitations that need to be taken into account. Firstly, the number of available studies was limited and future research to corroborate and extend the present findings is necessary. In view of the predominant qualitative nature of the topic under investigation which necessitated the qualitative review approach, future projects should focus on the quantification of the phenomenon. Experimental research in particular might help to determine the efficacy of different support measures highlighted in this review. Finally, this review was limited to a specific programme within a South African context and any generalisation must be treated with caution.

## Conclusions and recommendations

A CSP offers rich opportunities to CSNPs for professional learning, growth and development during the student-to-work transition period. However, findings from this study indicate that the CSP in South Africa is characterised by significant individual and contextual challenges, constraints, tensions as well as misalignments between theoretical knowledge provided at nursing education institutions and clinical practice. Improvements of the transition process are likely to be achieved through the development of more systematic support strategies. These strategies include structured orientation programmes which will serve as guidance for new nurses; authentic mentorship constructs wherein seasoned nurses are recruited to serve as mentors for newcomers; formal and informal support systems that create a safe and strong network for CSNPs; and education programmes such as peer support programmes that help facilitate the sharing of experience among CSNPs. Further recommendations include the development of mentorship workshops and training for seasoned nurses in order to provide emotional support to newly graduated nurses and aid retention (Chachula, Myrick & Yonge 2015). To tackle challenges linked to high workload, it is recommended that the Department of Health develop policies that ensure adequate and sufficient staffing in the rural and/or underserved communities.

Lastly, this review identifies a need for further research to (1) create more robust and evidence-based findings and (2) to test various potential approaches and strategies to empower CSNPs during this period of transition to aid learning, job satisfaction and retention of new graduates (Parker et al. 2014b; Pasila et al. 2017; Halpin et al. 2017).
